# A hypothesis is a liability

**DOI:** 10.1186/s13059-020-02133-w

**Published:** 2020-09-03

**Authors:** Itai Yanai, Martin Lercher

**Affiliations:** 1 Institute for Computational Medicine, NYU Langone Health, New York, NY 10016 USA; 2 Institute for Computer Science & Department of Biology, Heinrich Heine University, 40225 Düsseldorf, Germany

“ ‘When someone seeks,’ said Siddhartha, ‘then it easily happens that his eyes see only the thing that he seeks, and he is able to find nothing, to take in nothing. [...] Seeking means: having a goal. But finding means: being free, being open, having no goal.’ ” Hermann Hesse

There is a hidden cost to having a hypothesis. It arises from the relationship between night science and day science, the two very distinct modes of activity in which scientific ideas are generated and tested, respectively [1, 2]. With a hypothesis in hand, the impressive strengths of day science are unleashed, guiding us in designing tests, estimating parameters, and throwing out the hypothesis if it fails the tests. But when we analyze the results of an experiment, our mental focus on a specific hypothesis can prevent us from exploring other aspects of the data, effectively blinding us to new ideas. A hypothesis then becomes a liability for any night science explorations. The corresponding limitations on our creativity, self-imposed in hypothesis-driven research, are of particular concern in the context of modern biological datasets, which are often vast and likely to contain hints at multiple distinct and potentially exciting discoveries. Night science has its own liability though, generating many spurious relationships and false hypotheses. Fortunately, these are exposed by the light of day science, emphasizing the complementarity of the two modes, where each overcomes the other’s shortcomings.

## The gorilla experiment

Many of us recall the famous selective attention experiment, where subjects watch a clip of students passing a basketball to each other [3, 4]. If you have not seen it, we recommend watching it before continuing to read [5]. As you watch the two teams in action, your task is to count the number of passes made by the team in white. About halfway through, a person dressed up as a gorilla enters the foreground. The gorilla pauses in the center, pounding its chest with its fists, before exiting to the opposite side of the frame. Surprisingly, half of us completely miss the gorilla, as we are focused on counting passes, even though hardly anyone overlooks it when simply watching the clip without the assignment.

**Fig. 1:**
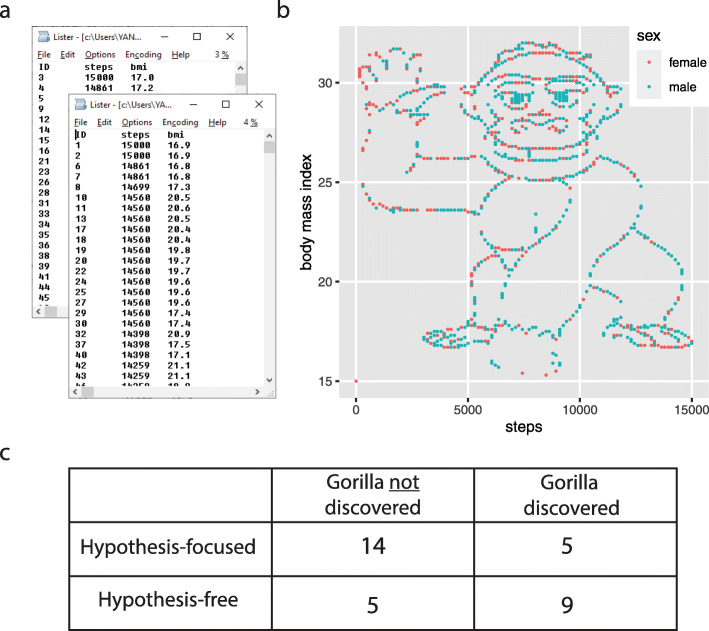
**a** An artificial dataset given to students with and without explicit hypotheses on the relationship between BMI and the steps taken on a particular day, for men and women. **b** A plot of the dataset. **c** The contingency table for students in the two groups (“hypothesis-focused,” “hypothesis-free”) that discovered the gorilla or not [6]

We wondered if a similar process occurs when we analyze a dataset. Would the mental focus on a specific hypothesis prevent us from making a discovery? To test this, we made up a dataset and asked students to analyze it [6]. We described the dataset as containing the body mass index (BMI) of 1786 people, together with the number of steps each of them took on a particular day, in two files: one for men, one for women (Fig. 1a). The students were placed into two groups. The students in the first group were asked to consider three specific hypotheses: (i) that there is a statistically significant difference in the average number of steps taken by men and women, (ii) that there is a negative correlation between the number of steps and the BMI for women, and (iii) that this correlation is positive for men. They were also asked if there was anything else they could conclude from the dataset. In the second, “hypothesis-free,” group, students were simply asked: What do you conclude from the dataset?

The most notable “discovery” in the dataset was that if you simply plotted the number of steps versus the BMI, you would see an image of a gorilla waving at you (Fig. 1b). While we teach our students the benefits of visualization, answering the specific hypothesis-driven questions did not require plotting the data. We found that very often, the students driven by specific hypotheses skipped this simple step towards a broader exploration of the data. In fact, overall, students without a specific hypothesis were almost *five* times more likely to discover the gorilla when analyzing this dataset (odds ratio = 4.8, *P* = 0.034, *N* = 33, Fisher’s exact test; Fig. 1c). At least in this setting, the hypothesis indeed turned out to be a significant liability.

## Not all who wander are lost

We typically acquire data with the expressed goal of testing a specific hypothesis. But as we have seen with the gorilla experiment, we are likely to miss other interesting phenomena as soon as we are in a mental mode of hypothesis testing. To account for this, we must consciously adopt a different mindset—one of exploration, where we look at the data from as many angles as possible. In this mode, we take on a sort of playfulness with the data, comparing everything to everything else. We become explorers, building a map of the data as we start out in one direction, switching directions at crossroads and stumbling into unanticipated regions.

Essentially, night science is an attitude that encourages us to explore and speculate. We ask: What could be hiding here? How would we lure it out? Night science may occur when we are most relaxed, such as when Friedrich Kekulé dreamingly looked into the fireplace in his study on an evening in 1862, until his mind formed the image of a molecular serpent biting its own tail—an image that he immediately converted into the hypothesis for the ring structure of benzene [7]. However, more often than not, night science may require the most acute state of mental activity: we not only need to make connections where previously there were none, we must do this while contrasting any observed pattern on an elaborate mental background that represents the expected. To see the discovery in our gorilla experiment, all that was needed was some notion of primate appearances. But when you roam the limits of the scientific knowns, you need a deep understanding of a field to even recognize a pattern or to recognize it as surprising. Different scientists looking at a given dataset will do this against a backdrop of subtly different knowledge and expectations, potentially highlighting different patterns. Looking is not the same as seeing, after all, and this may be why some of us may stumble upon discoveries in data that others have already analyzed.

## Patternicity or “just a correlation”?

“Correlation is not causation”—an aphorism that perhaps all scientists have heard at least once in their careers—warns of putting too much weight on mere covariation of two variables. Undoubtedly, a correlation between two features is not sufficient to infer a causal relationship. But some form of covariation is implied by a causal relationship, and hence, finding a previously hidden correlation may be the first glimpse of something new. We may then think of data exploration as the generator of correlations and patterns that can later be tested for causality.

One of the major facilitators of human intelligence is our minds’ ability to easily find patterns and connections—a tendency called patternicity by Michael Shermer [8]. Patternicity helps us in generating new night science ideas; it is the seed of many discoveries. On the flipside, patternicity makes us vulnerable to being fooled by randomness [9], when we mistakenly infer relationships between genuinely independent things (called apophenia). Clearly, spurious results will be generated during unguided explorations, and this generation of false starts is night science’s own liability.

Day science tempers this liability. In a sense then, correlations are the domain of night science, while causation is solidified by day science. Day science is the adult in the room, rigorously testing hypotheses. But despite its power, the day science mode is not amenable to generating the ideas in the first place. Only the night science realm, with its lack of specific hypotheses that blind us in day science, allows us to think freely in an exploratory fashion. Science relies on this back and forth between day and night, each overcoming the other’s shortcomings; we can let ourselves explore so freely in night science because we trust ourselves to check the generated hypotheses later, in day science.

## Fishing expeditions

In many scientific circles, one of the most condemning judgments about a project is to label it as “a fishing expedition”: an exploration of data that lacks even the pretense of a hypothesis. But as we argued above, such hypothesis-focused criticism misses a crucial point. Discoveries are not only unexpected, they are also undiscoverable without data. Provided that a dataset is carefully designed to be rich in information relevant to a specific field, initially hypothesis-free night science explorations are a systematic way to generate hypotheses, a way that is not only powerful, but, in our opinion, also beautiful.

Many discoveries that we read about came out of projects that were originally devised as fishing expeditions or that turned into such after the original hypothesis had to be abandoned. But we rarely hear about this historical aspect, because a tale about a logically made hypothesis and then tested in rigorous day science makes for a much better story and because these are the kind of stories that editors and reviewers like to read. We know this from hearsay about many works of valued colleagues, but we know it best from our own publications. For example, Tin Pang assembled a dataset connecting genotypes and phenotypes across the evolution of the *E. coli* clade, looking for further support for our hypothesis of bacterial evolution through stepwise niche expansion [10]. But analyzing the data, we found something much more interesting [11]: none of the more than 3000 detectable metabolic innovations in the history of *E. coli* required more than a single horizontal gene transfer! Another project, led by then-graduate student Michal Levin, involved the collection of a gene expression dataset of embryogenesis in 5 species of worms, assembled based on the idea that it might reveal gene regulatory networks. Analyzing the dataset instead led us to find a distinctive developmental stage, which we inferred to be the nematode phylotypic stage [12].

## Keep exploring and carry on

One thing we have learned from decades of exploratory data analysis: do not give up on a dataset. If it does not support your original hypothesis, it likely contains hints at alternative, possibly even more interesting phenomena. And if the data supports your original hypothesis, still keep exploring beyond. If the dataset has been designed and assembled well, there are likely additional discoveries to be made. These cannot be expected to emerge after just a first look. They will take time to unfold. It is not well appreciated, but the truth is that one never really finishes to analyze a dataset. You just decide to stop and move on at some point, leaving some things undiscovered. Because night science demands a highly creative state, it is not surprising that this process mirrors the situation in the arts as described by the poet Paul Valéry in 1933: “un ouvrage n’est jamais achevé . . . mais abandonné” (“a work is never finished, only abandoned”).

In line with the premise of this article, we of course had to explore our own gorilla experiment dataset beyond our original hypothesis—that hypotheses may prevent discoveries. We indeed found hints at something else: hypotheses may also lead you to give up on your data prematurely. The students who had a hypothesis to test were more than twice as likely to not even attempt the exercise or to give up after the first initial steps. While this difference is not statistically significant (odds ratio = 2.15, *P* = 0.21, *N* = 44, Fisher’s exact test), it suggests further day science experiments. Maybe we will keep our students motivated in science by providing more opportunities for data exploration and discovery.

In sum, keep your mind open when working with data. Think about the particular dimensionality of your dataset and study the variation across these. Consider what the variation along these dimensions may reflect, and try to connect that to aspects beyond the dataset. By asking what other dimensions could be integrated to explain the observed variation, you are positioning yourself for a discovery. Let your fantasies run wild to generate classes of hypotheses that would leave traces in the data. There could be gorillas hiding in there.

## References

[1] Yanai I, Lercher M (2019). Night science. Genome Biol.

[2] Jacob F. The statue within: an autobiography. New York: Cold Spring Harbor Laboratory Press; 1988.

[3] Simons DJ, Chabris CF (1999). Gorillas in our midst: sustained inattentional blindness for dynamic events. Perception.

[4] Chabris C, Simons D. The invisible gorilla: and other ways our intuition deceives us. New York: Random House; 2009.

[5] https://www.youtube.com/watch?v=vJG698U2Mvo. Accessed 16 Jul 2020.

[6] Yanai I, Lercher M. Selective attention in hypothesis-driven data analysis. BioRxiv. 2020. 10.1101/2020.07.30.228916.

[7] Schultz G (1890). Feier der Deutschen Chemischen Gesellschaft zu Ehren August Kekulé’s. Ber Dtsch Chem Ges.

[8] Shermer M (2008). Patternicity. Sci Am.

[9] Taleb NN. Fooled by randomness: the hidden role of chance in life and in the markets. New York: Random House; 2005.

[10] Szappanos B, Fritzemeier J, Csörgő B (2016). Adaptive evolution of complex innovations through stepwise metabolic niche expansion. Nat Commun.

[11] Pang TY, Lercher MJ (2019). Each of 3,323 metabolic innovations in the evolution of *E. coli* arose through the horizontal transfer of a single DNA segment. Proc Natl Acad Sci U S A.

[12] Levin M, Hashimshony T, Wagner F, Yanai I (2012). Developmental milestones punctuate gene expression in the Caenorhabditis embryo. Dev Cell.

